# Diet in Enteric Fever

**Published:** 1893-08

**Authors:** 


DIET IN ENTERIC FEVER.—From one of our valuable exchanges we obtain the following hints on diet in enteric fever. The writer, Dr. C. M. Ernst, says that milk is no doubt the best food where it suits the patient; and that three, or to the outside four, pints of liquid food is sufficient nourishment for an adult, and that proportionately less should be given to children. Great care is necessary when the patient becomes convalescent, as relapses are so frequent and, at the same time, so easy. The change in diet is very gradual, at first an increase in the quantity and then thickening the milk with corn flour or arrowroot; then, after a few days a lightly boiled egg, then a little bread, then fish, and so on. The temperature should be carefully watched in order to detect any rise at once.—*Ex.*

